# Enhanced Amygdala-Striatal Functional Connectivity during the Processing of Cocaine Cues in Male Cocaine Users with a History of Childhood Trauma

**DOI:** 10.3389/fpsyt.2018.00070

**Published:** 2018-03-12

**Authors:** Anne Marije Kaag, Liesbeth Reneman, Judith Homberg, Wim van den Brink, Guido A. van Wingen

**Affiliations:** ^1^Department of Developmental Psychology, University of Amsterdam, Amsterdam, Netherlands; ^2^Departement of Psychiatry, Academic Medical Centre, Amsterdam, Netherlands; ^3^Amsterdam Brain and Cognition, University of Amsterdam, Amsterdam, Netherlands; ^4^Departement of Radiology and Nuclear Medicine, Academic Medical Centre, Amsterdam, Netherlands; ^5^Donders Institute for Brain, Cognition, and Behaviour, Radboud University, Medical Centre, Nijmegen, Netherlands

**Keywords:** cocaine addiction, anxiety, childhood trauma, functional connectivity, ventral striatum, amygdala, dorsal medial prefrontal cortex, negative reinforcement

## Abstract

**Background and aims:**

Childhood trauma is associated with increased levels of anxiety later in life, an increased risk for the development of substance use disorders, and neurodevelopmental abnormalities in the amygdala and frontostriatal circuitry. The aim of this study was to investigate the (neurobiological) link among childhood trauma, state anxiety, and amygdala-frontostriatal activity in response to cocaine cues in regular cocaine users.

**Methods:**

In this study, we included 59 non-treatment seeking regular cocaine users and 58 non-drug using controls. Blood oxygenation level-dependent responses were measured using functional magnetic resonance imaging while subjects performed a cue reactivity paradigm with cocaine and neutral cues. Psychophysiological interaction analyses were applied to assess functional connectivity between the amygdala and other regions in the brain. Self-report questionnaires were used to measure childhood trauma, state anxiety, drug use, drug use severity, and craving.

**Results:**

Neural activation was increased during the presentation of cocaine cues, in a widespread network including the frontostriatal circuit and amygdala in cocaine users but not in controls. Functional coupling between the amygdala and medial prefrontal cortex was reduced in response to cocaine cues, in both cocaine users and controls, which was further diminished with increasing state anxiety. Importantly, amygdala-striatal connectivity was positively associated with childhood trauma in regular cocaine users, while there was a negative association in controls. At the behavioral level, state anxiety was positively associated with cocaine use severity and craving related to negative reinforcement.

**Conclusion:**

Childhood trauma is associated with enhanced amygdala-striatal connectivity during cocaine cue reactivity in regular cocaine users, which may contribute to increased habit behavior and poorer cognitive control. While we cannot draw conclusions on causality, this study provides novel information on how childhood trauma may contribute to the development and persistence of cocaine use disorder.

## Introduction

Substance use disorder (SUD) is characterized by compulsive drug use, loss of control in limiting intake, and emergence of a negative emotional state when access to the drug is denied (1, 2). Through the process of negative reinforcement, negative emotional states are suggested to induce craving and drug taking behavior in substance-dependent individuals (1–7). Supporting the role of stress in the development of SUD, childhood trauma (as an indicator of early life stress) is associated with a greater likelihood of developing an SUD (8–15). More specifically, a history of childhood trauma is associated with an increased risk to transition from recreational to compulsive substance use (16), reduced abstinence motivation (17), and an increase in withdrawal symptoms during early abstinence (18). In addition to childhood trauma, acute negative emotional states such anxiety have consistently been associated with SUD (19–23). Altogether, childhood trauma and negative emotional states are suggested to be involved in the development and persistence of SUD, but the neural pathways that underlie this relationship have so far been unexplored.

However, extensive evidence that childhood trauma induces neurodevelopmental changes within the prefrontal cortex (24–28), striatum (26, 29–31), and the amygdala (26, 28, 32, 33). While the frontostriatal circuit plays a crucial role in drug-reward anticipation and inhibitory control (34, 35), it is the amygdala that has been suggested to underlie negative reinforcement in SUD (34, 36). Several cue reactivity studies have indeed demonstrated increased amygdala activation in response to substance-related cues, supporting the role of the amygdala in drug-related behavior (37–39). However, we have recently demonstrated that the amygdala in regular cocaine users is also hyperresponsive to negative emotional stimuli in general (40, 41), which is suggested to be normalized by substance intake (42). However, it is still unclear how the amygdala modulates frontostriatal processing of drug-related cues and how this is related to childhood trauma and negative emotional states. The aim of this study is therefore to further explore this relationship in a sample of male regular cocaine users. Because most previous research focused on male cocaine users, and cocaine use is more than twice as prevalent among males then females (43), we focused this research on male cocaine users only.

Previous studies have demonstrated that the amygdala receives input from several prefrontal regions and in turn projects to widespread striatal domains (44). In this way, the amygdala can modulate striatal output during reward learning and performance (45, 46). By using psychophysiological interaction (PPI) analyses to identify task-related changes in functional connectivity (47), it has been demonstrated that impaired functional coupling between the amygdala and prefrontal cortex is negatively associated with cognitive control over negative emotions (48, 49), whereas impaired functional coupling between the amygdala and striatum is negatively associated with increased risk seeking behavior (50). In the current study, we used PPI (regression) analyses to investigate how cocaine cues alter the functional connectivity between the amygdala and frontostriatal network and how this is related to childhood trauma and state anxiety, as an index of negative emotional states. On the basis of previous research, we expected that cocaine cues would impair the functional connectivity between the amygdala and frontostriatal circuit and that this would deteriorate depending on the level of childhood trauma and state anxiety.

## Subjects and Methods

### Participants

A total of 66 non-treatment seeking male regular cocaine users and 66 non-drug using controls were included in this study. A total of 7 CU and 8 HC were excluded because of MRI artifacts or missing values of the relevant questionnaires, resulting in the inclusion of 58 controls and 59 cocaine users in the analyses. All participants were males (aged 18–50 years) recruited through local advertisement in the greater Amsterdam area in the Netherlands. Inclusion criteria for cocaine users were snorting cocaine at least once per week for a minimum period of 6 months. General exclusion criteria were major medical or neurological disease, lifetime history of psychotic or bipolar disorder or the presence of contraindications to MRI scanning (e.g., claustrophobia or implanted ferromagnetic objects), the use of antidepressants and/or antipsychotics, and a positive urine screening on opioids. Control subjects were also excluded if they met DSM-IV criteria for lifetime substance abuse or dependence or currently took any psychotropic medications other than antidepressants or antipsychotics. The study was approved by the Ethical Review Board of the Academic Medical Centre of the University of Amsterdam, the Netherlands. All subjects gave written informed consent.

### General Procedure

After participants arrived at the research center, they were informed about all study procedures after which they gave written informed consent. After they completed the demographic and clinical assessment, they were asked to provide a urine sample to test for the presence of cocaine, opioids, amphetamines, and alcohol metabolites. After the MRI scan, all participants were asked to validate the neutral and cocaine pictures on a computer.

### Clinical and Demographic Assessment

Participants were psychiatrically evaluated with the Mini-International Neuropsychiatric Interview [MINI (51)] on the presence of lifetime substance abuse or dependence, depressive episodes, and anxiety disorders. Childhood trauma was quantified using the Dutch version of the Brief Childhood Trauma Questionnaire [CTQ (52)]. The CTQ consists of five subscales, which together make a total score of childhood trauma severity. For each subscale, clinical cutoff scores can be used to differentiate between none to low and moderate to severe emotional abuse (cutoff ≥ 13), physical abuse (cutoff ≥ 10), sexual abuse (cutoff ≥ 8), emotional neglect (cutoff ≥ 15), and physical neglect (cutoff ≥ 10) (53). State anxiety, as a measure of negative emotional states, was quantified using the state-trait anxiety inventory [STAI (54)]. A cutoff point of 39–40 is normally used for clinically significant symptoms of state anxiety (55–57). Depressive symptoms were further assessed using the Beck depression inventory (58). Premorbid intelligence (IQ) was assessed using the Dutch Adult Reading Test [DART (59)]. Cocaine use, in addition to alcohol, cannabis, and NDMA use in the 6 months before study inclusion was quantified using the timeline follow back procedure (60). The drug use disorder identification test (DUDIT) was used to assess cocaine use severity (61). Finally, the desire for drug questionnaire (DDQ) was used to measure the desire and intention to use cocaine (DDQ-desire), the use of cocaine to relief negative states (DDQ-negative reinforcement), and the perceived control over cocaine use (DDQ-control) (62).

### Experimental Paradigm

In this study, we used a modified version of the event-related cue reactivity paradigm previously implemented by Cousijn et al. (63), using full-color cocaine-related pictures (*n* = 46), neutral control pictures (*n* = 46), and target pictures (*n* = 12). Cocaine pictures were photos of cocaine and individuals snorting cocaine. Neutral control pictures were photos of individuals and objects visually matched to the cocaine pictures on color, composition, and the type of gesture (passive or active), but without any referral to cocaine or cocaine use. Target pictures were photos of animals. The pictures are available on request. Participants were asked to pay attention to the pictures. To ensure maintained attention, they were instructed to press a key on a response box when they saw the animal. Each image was presented for 4 s and was preceded by a fixation-cross that lasted on average 4 s, jittered between 2 and 6 s. The task had a total duration of approximately 14 min. The cocaine, control, and animal pictures were presented in a same semi-random order (with a maximum of three images of the same category in a row) for each participant. Images were projected on a screen viewed through a mirror attached to the MRI head coil. Craving was assessed inside the MRI scanner, at baseline and at the end of the experimental paradigm, using a visual analogue scale ranging from 0 (not at all) to 10 (extremely), asking “How much do you crave for cocaine right now?”

### Validation of the Cocaine and Neutral Cues

After the MRI scan, all participants performed an image-rating task outside the MRI scanner. In this task, they had to rate the images that were presented inside the MRI scanner on how much it induced craving and arousal, using a VAS, ranging from 1 to 10.

### Behavioral Data Analysis

Group differences in demographic and clinical characteristics were assessed using independent samples *t*-tests or non-parametric tests when appropriate, using SPSSv22 (Statistical Package for the Social Science). Data are presented as mean ± SD or medians ± interquartile range (ICQ), where appropriate.

Repeated measures (RM) ANOVAs, with stimulus type as RM, group as an independent variable and stimulus rating as a dependent variable, were applied to test the interaction between group and stimulus type on the rating of the stimuli.

Within cocaine users, partial correlations were computed between state anxiety (controlled for childhood trauma) or childhood trauma (controlled for state anxiety) and cocaine use severity (DUDIT scores), monthly cocaine use, and the desire for cocaine (mean scores on the DDQ-desire, DDQ-negative reinforcement and DDQ-control).

Changes in self-reported craving during the cue reactivity paradigm were tested using a RM ANOVA, with time (prescanning or postscanning) as the RM. In addition, childhood trauma, state anxiety, and its interaction term were entered in the model to test the relation between cue-induced craving and these variables.

### Functional Magnetic Resonance Imaging (fMRI) Data Acquisition and Analysis

Images were acquired on a 3.0-T Achieva full-body scanner (Philips Medical Systems, Best, the Netherlands) using a 32 channel SENSE head coil. Echo planar images were taken covering the whole brain, with a total of 37 ascending axial slices (3 mm × 3 mm × 3 mm voxel size; slice gap 3 mm; TR/TE 2,000 ms/28 ms; matrix 80 × 80). Also a T1-3D high-resolution anatomical scan (TR/TE 8.2/3.7; matrix 240 × 187; 1 mm × 1 mm × 1 mm voxel; transverse slices) was taken. fMRI data were analyzed using SPM8. Preprocessing included realignment, slice-time correction, co-registration of the structural and functional scans, normalization to MNI space based on the segmented structural scan, and smoothing with a Gaussian kernel of 8 mm full-width at half maximum. First-level models included separate regressors for the cocaine cues, control cues, and targets. These regressors were convolved with the canonical hemodynamic response function. Six realignment parameters were included as regressors of no interest. A high pass filter (1/128 Hz) was included in the first-level model to correct for low-frequency signal drift.

The contrasts for cocaine and control cues were entered in a second level full-factorial design. First, we tested for a group by stimulus type interaction effect. Second, we tested a group by stimulus type by childhood trauma (CTQ) by STAI-state interaction effect, by adding the total CTQ scores and STAI-state *z*-scores and their interaction term as a covariate in the second-level model.

To investigate whether and how cocaine cues alter the functional coupling between the amygdala and other brain regions, we used generalized psychophysiological interaction analysis (64) with the left and right amygdala as seed regions. This type of analysis allows investigating changes in functional connectivity with a seed region (in this case the amygdala) related to a certain psychological variable (in this case, the presentation of either a cocaine-related or neutral stimulus). The time series of the first eigenvariate of the blood oxygenation level-dependent signal were temporally filtered, mean corrected, and deconvolved to generate the time series of the neuronal signal for the left and right amygdala for each individual subject. The interaction term—PPI—was computed by multiplying the time series from the psychological regressors with this physiological variable. First, we tested a group by stimulus type interaction effect on functional connectivity. To assess how differences in functional connectivity during cue reactivity are related to state anxiety, childhood trauma, or the combination of both, the total CTQ scores and STAI-state *z*-scores and their interaction term were entered as covariates in the PPI analysis.

Whole-brain second-level analyses were family-wise error (FWE) rate corrected on cluster level (*p* < 0.05), with an initial height threshold on voxel level of *p* < 0.001. A small-volume correction was applied for the amygdala and ventral striatum (*p* < 0.05) because of the *a priori* role in cue reactivity. These region of interest analyses were few corrected at peak level, and only clusters with a minimum cluster size of 10 are reported. The amygdala was defined based on the automatic anatomical labeling (AAL) as implemented in SPM8. Because the AAL atlas does not include the VS, the VS was defined as the nucleus accumbens from the Harvard-Oxford subcortical structure probability atlas. For all analyses, only in case of a significant effect, the appropriate within-group analyses were performed. In case of a non-significant interaction effect, only the main effects are reported.

## Results

### Demographic and Clinical Characteristics

For all demographic and clinical characteristics, see Table 1. All participants were from north-west European descent. Groups were of similar age (F_1,115_ = 0.01, *p* = 0.92), but cocaine users had significantly lower IQ scores (F_1,115_ = 6.82, *p* = 0.010). Cocaine users had significantly higher scores on the CTQ compared to controls (F_1,115_ = 19.97, *p* < 0.001). More specifically, based on the clinical cutoff scores (53), cocaine users reported a significantly higher prevalence of moderate to severe childhood trauma on all categories (emotional and sexual abuse, emotional and physical neglect) except physical abuse (χ^2^ = 24.5, *p* < 0.001). The total STAI-state score was significantly higher in cocaine users compared to controls (F_1,115_ = 19.99, *p* < 0.001). The prevalence of clinically significant symptoms of state anxiety was 34% (*n* = 20) in cocaine users and 10.3% (*n* = 6) in non-drug using controls. However, the lifetime prevalence of an anxiety disorder did not differ between groups. As expected, scores for childhood trauma and state anxiety were significantly correlated (*r* = 0.276 and *p* = 0.034). Lifetime prevalence of depressive episodes (χ^2^ = 13.45, *p* < 0.001) and BDI scores (F_1,113_ = 58.5, *p* < 0.001) were also higher among cocaine users compared to non-drug using controls. Moreover, BDI scores were significantly and positively correlated to state anxiety (*r* = 0.53, *p* < 0.001).

**Table 1:** Demographic and clinical information.

	Controls (*n* = 58)	Cocaine users (*n* = 59)	*p* Value
Age	30.5 ± 8.1	31.4 ± 7.6	n.s.
IQ	104.7 ± 9.0	100.2 ± 8.4	0.01
Childhood trauma—total score	33.5 ± 7.5	42 ± 14	
Childhood trauma—number of maltreatment categories			<0.001
0 maltreatment categories	77.6% (*n* = 45)	37.3% (*n* = 22)	
1 maltreatment categories	10.3% (*n* = 6)	33.9% (*n* = 20)	
2 maltreatment categories	12.1% (*n* = 7)	13.6% (*n* = 8)	
3 maltreatment categories	0%	11.9% (*n* = 7)	
4 maltreatment categories	0%	0%	
5 maltreatment categories	0%	3.4% (*n* = 2)	
Childhood trauma—types maltreatment (moderate/severe)			
Emotional abuse	3.4% (*n* = 2)	16.9% (*n* = 10)	0.029
Physical abuse	1.7% (*n* = 1)	5.1% (*n* = 3)	n.s.
Sexual abuse	1.7% (*n* = 1)	13.6% (*n* = 8)	0.017
Emotional neglect	13.8% (*n* = 8)	35.6% (*n* = 21)	0.006
Physical neglect	13.8% (*n* = 8)	42.4 (*n* = 25)	0.001
State anxiety (total score)	28 ± 8	35 ± 16	<0.001
State anxiety (prevalence of clinically significant symptoms)	10.3% (*n* = 6)	34% (*n* = 20)	
Lifetime prevalence of anxiety disorder	1.7% (*n* = 1)	6.8% (*n* = 4)	
Beck Depression Inventory	2.75 ± 3.45	10.62 ± 6.97	<0.001
Lifetime prevalence of major depressive disorder	6.8% (*n* = 4)	34.4% (*n* = 20)	<0.001
Weekly alcohol intake	3 ± 5.5	20 ± 22.5	
Lifetime alcohol abuse or dependence (DSM-IV)	0%	32.2% (*n* = 19)	
Cocaine use—g/month	–	7.33 ± 6.08	
Cocaine use—days/month	–	8.50 ± 5.67	
Cocaine use—duration (years)	–	6.00 ± 12.00	
Cocaine use—age of onset	–	19.00 ± 4.00	
Cocaine use severity	–	18.4 ± 5.83	
Lifetime cocaine abuse or dependence (DSM-IV)	–	93% (*n* = 56)	
Desire for cocaine use questionnaire			
Desire	–	2 ± 2	
Negative reinforcement	–	2.5 ± 2	
(Loss of) control	–	3.80 ± 1.51	
Weekly cannabis use	–	38.9% (*n* = 23)	
Lifetime cannabis abuse or dependence (DSM-IV)	–	28.8% (*n* = 17)	
MDMA use in the last 6 months	–	49.1% (*n* = 29)	
Lifetime MDMA/XTC abuse or dependence (DSM-IV)	–	8.5% (*n* = 5)	

Cocaine users used on average 7.33 ± 6.08 g of cocaine per month, on 8.50 ± 5.67 days, were using regularly for 6.00 ± 12.00 years and started using at 19.00 ± 4.00 years of age. The majority (93%) of the cocaine users met DSM-IV criteria for lifetime cocaine abuse or dependence and reported a mean total DUDIT score of 18.4 ± 5.83. Mean scores on the DDQ subscales were 2 ± 2 for DDQ-desire, 2.5 ± 2 for 2 DDQ-negative reinforcement, and 3.80 ± 1.51 for DDQ-control. Of all cocaine users, 39.7% (*n* = 23) used cannabis on a weekly basis or more, and 28.8% (n = 17) met the DSM-IV criteria for lifetime cannabis abuse or dependence. Moreover, 50% (*n* = 29) used MDMA in the last 6 months at least once and 8.5% (*n* = 5) met the DSM-IV criteria for lifetime XTC/MDMA abuse or dependence. Finally, 35.6% (*n* = 21) consumed more than 21 units of alcohol per week and 32.2% (*n* = 19) met the DSM-IV criteria for lifetime alcohol abuse or dependence. The urine screens revealed that none of the cocaine users scored positive on alcohol or opioids, 4 scored positive on amphetamine metabolites, and 29 scored positive on cocaine metabolites. However, because cocaine and amphetamine can be detected in urine of regular cocaine users up to 12 days after use, individuals with a positive urine screening were not excluded from the analyses.

State anxiety (controlled for childhood trauma) was significantly correlated with cocaine use severity (DUDIT: *r* = 0.38, *p* = 0.0023) and craving related to negative reinforcement (DDQ-negative reinforcement: *r* = 0.39, *p* = 0.002) but not with monthly cocaine use or craving related to desire and loss of control. There was no significant correlation between childhood trauma and cocaine use, cocaine use severity, or craving for cocaine.

### Cue-Induced Craving and Postexperiment Validation of Cocaine Cues

Repeated measures ANOVAs showed that there was a significant group by time (before and after cue reactivity) interaction effect on craving (F_1,112_ = 18.78, *p* < 0.001). A within-group follow-up tests confirmed that only in cocaine users, and not in non-drug using controls, craving for cocaine significantly increased during the cue reactivity task (F_1,57_ = 22,17, *p* < 0.001). Within cocaine users, there was no statistically significant interaction with childhood trauma or state anxiety.

Postscanning validation of the neutral and cocaine cues has been assessed in 41 cocaine users and 43 controls. RM ANOVAs demonstrated that there was a significant group by stimulus type interaction effect on craving (F_1,82_ = 68.78, *p* < 0.001) and arousal (F_1,70_ = 31.58, *p* < 0.001). Within-group follow-up analyses demonstrated that cocaine cues were rated as significantly more arousing than neutral cues in cocaine users (F_1,35_ = 43.63, *p* < 0.001; mean rating cocaine cues: 4.35 ± 1.97; mean rating neutral cues: 1.97 ± 1.10) but not in controls (F_1,35_ = 0.28, *p* = 0.60; mean rating cocaine: 1.68 ± 1.00; mean rating neutral cues: 1.83 ± 1.23). Similarly, cocaine cues were rated as significantly more craving inducing than neutral cues in cocaine users (F_1,40_ = 62.43, *p* < 0.001; mean rating cocaine cues: 4.56 ± 2.17; mean rating neutral cues: 1.86 ± 1.13) but not in controls (F_1,42_ = 3.44, *p* = 0.07; mean rating cocaine cues: 1.07 ± 0.22; mean rating neutral cues: 1.21 ± 0.57). These analyses confirm that cocaine pictures (and not neutral pictures) elicited strong feelings of craving and arousal in cocaine users only.

### Differences in Neural Activation Related to Cocaine Cues

**Figure 1:**
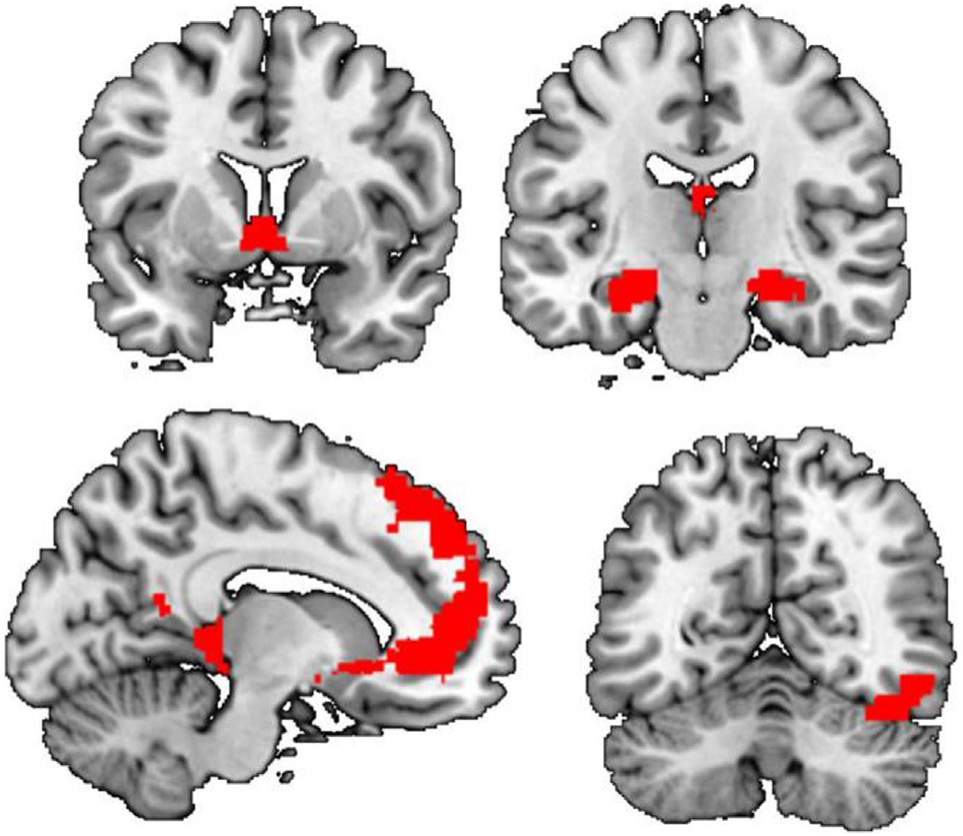
Main effect of cue reactivity. Compared to controls, cocaine users show enhanced cue reactivity within the dorsal and ventral anterior cingulate cortex, the bilateral ventral striatum, the left amygdala and right hippocampus/parahippocampal gyrus, and right occipital cortex.

Whole-brain analysis showed that there were significant group by stimulus type interaction effects. Compared to neutral cues, cocaine cues elicit greater activation in a wide range of brain regions in cocaine users compared to control (Figure 1; Table 2). These regions included the left orbital and bilateral superior frontal cortex, the left and right hippocampus, the right occipital cortex, and the bilateral nucleus accumbens. Within-group analysis showed that, in cocaine users, cocaine cues elicited greater activation compared to neutral cues, in various brain regions including the bilateral nucleus accumbens, the bilateral ventromedial and dorsomedial prefrontal cortex, and the right (para)hippocampus. There were no brain regions more strongly activated by neutral cues compared to cocaine cues in this group. Nor was there a significant stimulus effect in the non-drug using controls. These results demonstrate that cocaine cue reactivity is specific to cocaine users.

**Table 2:** Cue reactivity group by stimulus type interaction effect.

	Cluster size # voxels	Cluster *p* value	Voxel ***z*** value	Peak voxel MNI coordinates			Voxel region
**Cocaine > neutral**							
Cocaine users^b^	3,377	<0.001	>9.99	−2	48	−10	L Medial orbital frontal cortex
			7.36	0	36	−10	R Medial orbital frontal cortex
			6.19	−16	42	48	L Superior frontal gyrus
			5.37	−4	36	4	L Anterior cingulate gyrus
			4.18	−24	26	50	L Middle frontal gyrus
	344	0.002	7.65	18	−8	−18	R Parahippocampal gyrus
			6.87	22	−20	−14	R Hippocampus
	792	<0.001	7.5	56	−60	−12	R Inferior temporal gyrus
			7.12	24	−98	−2	R Inferior occipital gyrus
			6.57	46	−54	−20	R Fusiform gyrus
			6.31	46	−54	−26	R Cerebellum
			4.91	34	−82	8	R Middle occipital gyrus
			4.27	34	−74	−18	R Fusiform gyrus
	60	0.002	6.45	−4	6	−6	L Nucleus accumbens^a^
	20	0.006	5.64	6	6	−8	R Nucleus accumbens^a^
	46	<0.001	7.70	−20	−8	−16	L amygdala^a^
Controls^b^	No significant clusters						

**Neutral > cocaine**							
Cocaine users^b^	No significant clusters						
Controls^b^	No significant clusters						

*All results were p < 0.05, cluster level family-wise error corrected with an initial height threshold of p = 0.001 uncorrected*.

*^a^Peak value, corrected for the volume of the amygdala or nucleus accumbens, p_peak voxel_ < 0.05*.

*^b^Only those regions that show a significant group by stimulus type interaction effect are reported*.

Whole-brain regression analysis demonstrated that there was a significant group by stimulus type by childhood trauma by state anxiety interaction effect in the left precuneus, the left posterior cingulate cortex, and the left calcarine cortex. Within-group analysis demonstrated that this effect was significant only in non-drug using controls. All the other main and interaction effects were not significant.

### Differences in Functional Connectivity during Cocaine Cue Reactivity

**Figure 2:**
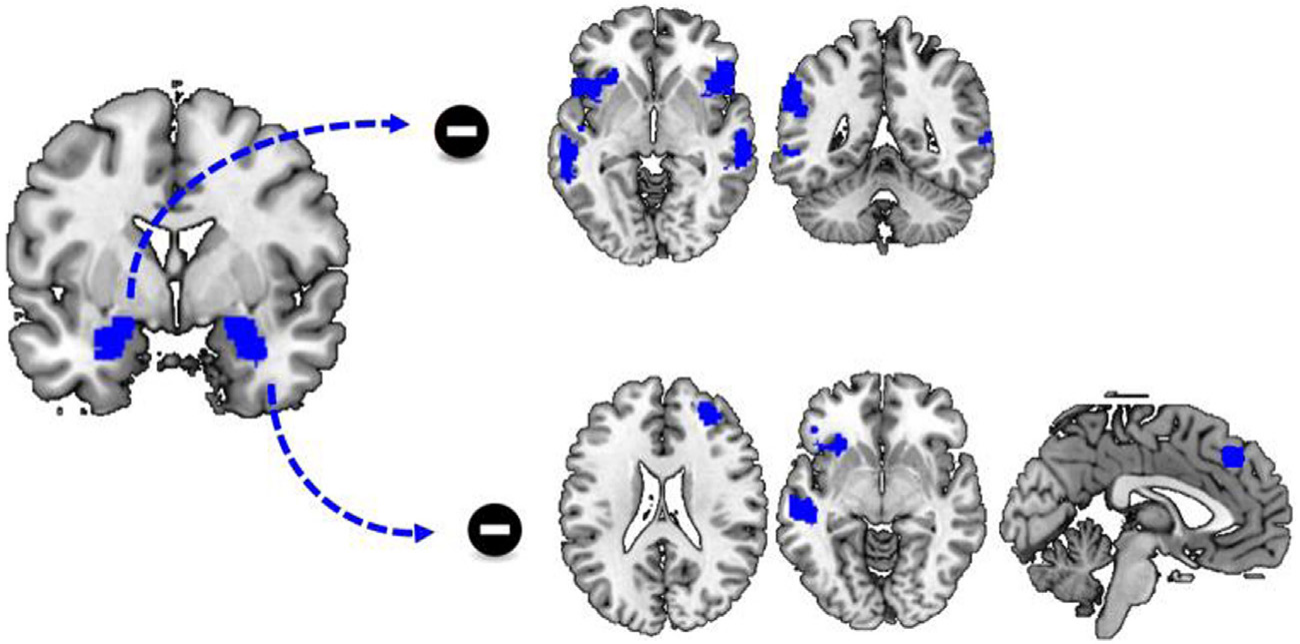
Differences in functional connectivity during cue reactivity. Functional connectivity between the left and right amygdala and a variety of brain regions is significantly reduced during the processing of cocaine cues compared to neutral cues. This includes functional connectivity between the left amygdala and the bilateral insula, inferior frontal cortex and the temporal cortex as well as functional connectivity between the right amygdala and the left insula, inferior frontal cortex and dorsomedial frontal cortex.

To assess how cocaine cues altered amygdala connectivity, we performed a PPI analysis. There was no significant group by stimulus type interaction effect on functional connectivity with the left amygdala as seed region. However, there was a significant main effect of stimulus type as there was a stronger functional connectivity of the left amgydala to a variety of other regions during the presentation of neutral cues, compared to the presentation of cocaine cues. These regions included bilateral middle and superior temporal cortex, insula, and inferior frontal cortex (Table 3; Figure 2).

**Table 3:** Differences in amygdala connectivity during the processing of cocaine and neutral cues (in cocaine users).

	Cluster size # voxels	Cluster *p* value	Voxel*z* value	Peak voxel MNI coordinates			Voxel region
**Left amygdala**							
Neutral > cocaine	792	<0.001	4.95	−60	−24	−4	L Middle temporal gyrus
			4.07	−46	−18	−8	L Superior temporal gyrus
	570	<0.001	4.77	−30	22	−8	L Insula
			4.15	−52	18	2	L Inferior frontal gyrus
	880	<0.001	4.39	54	−16	−12	Right middle temporal gyrus
			4.36	60	−20	−4	R Superior temporal gyrus
	774	<0.001	4.13	56	18	6	R Inferior frontal gyrus
			3.72	44	18	2	R Insula
			3.31	60	6	10	R Rolandic operculum
	309	0.008	4.04	−58	−46	34	L Supramarginal gyrus
			3.98	−60	−48	38	L Inferior parietal gyrus
Cocaine > neutral	No significant clusters						

**Right amygdala**							
Neutral > cocaine	324	0.003	4.42	−28	24	−6	L Insula
			3.6	−44	26	−10	L Inferior frontal gyrus
	386	0.001	4.39	−48	−22	−8	L Middle temporal gyrus
	209	0.023	4.12	34	44	22	L Middle frontal gyrus
			3.27	22	52	24	R Superior frontal gyrus
			3.23	30	36	30	R Middle frontal gyrus
	195	0.03	4.04	−6	34	44	L Medial frontal gyrus
	324	0.003	3.63	6	42	40	R Medial frontal gyrus
Cocaine > neutral	No significant clusters						

*All results were p < 0.05, cluster level family-wise error corrected with an initial height threshold of p = 0.001 uncorrected*.

*There was no significant group by stimulus type interaction effect on functional connectivity*.

Similar to functional connectivity with the left amygdala as seed region, there was no significant group by stimulus type interaction effect on functional connectivity with the right amygdala as seed region. However, there was a significant effect of stimulus type, as there was a stronger functional connectivity of the right amygdala to a variety of regions during the presentation of neutral cues, compared to the presentation of cocaine cues. These regions included the left insula, the left inferior and middle frontal cortex, the right superior and middle frontal cortex, and the bilateral superior medial frontal cortex and the left middle temporal cortex (Table 3; Figure 2).

#### The Relation between Functional Connectivity and Childhood Trauma

**Figure 3:**
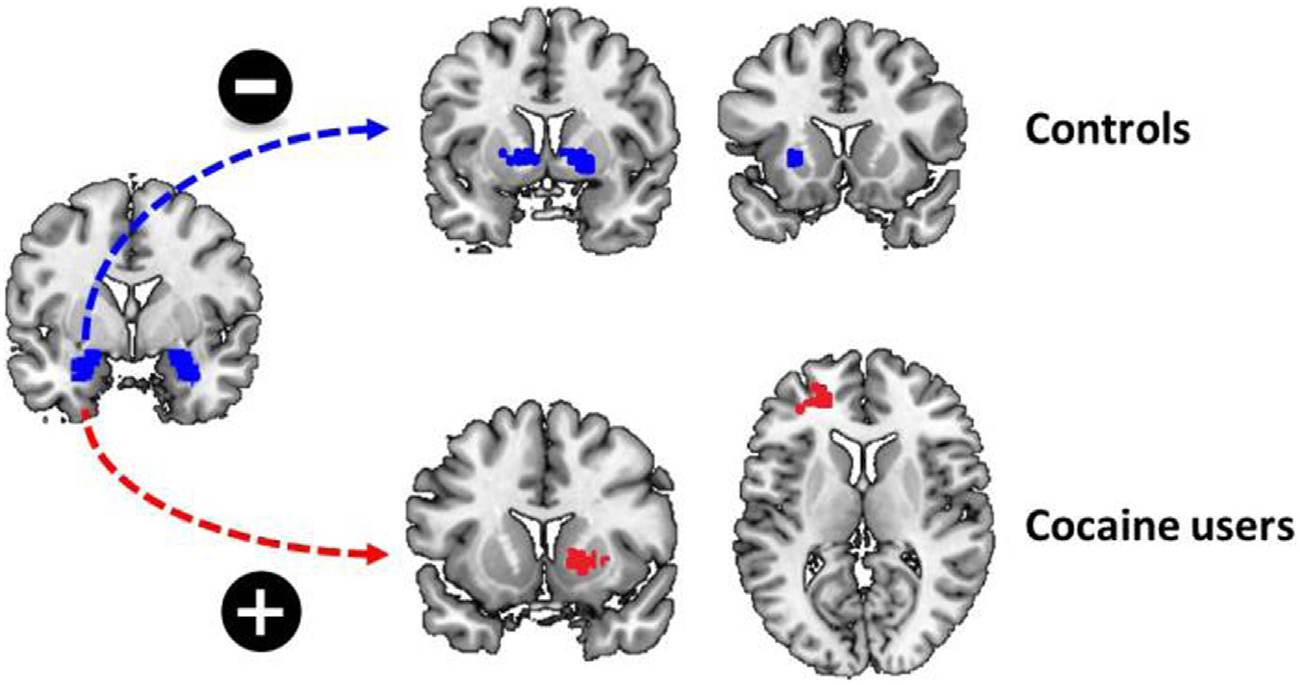
Childhood trauma and functional connectivity. There was a significant group by childhood trauma interaction on functional connectivity between the left amygdala and the dorsal striatum. While childhood trauma was negatively correlated with functional connectivity between the left amygdala and bilateral dorsal striatum in non-drug using controls, childhood trauma was positively correlated with functional connectivity between the left amygdala and left middle frontal cortex and right dorsal striatum.

There was a significant group by stimulus type by childhood trauma interaction effect on functional connectivity between the left amygdala and a variety of brain regions during cocaine cue reactivity. These regions included the left putamen, the right superior motor area, and the right middle cingulate cortex. Within-group analyses demonstrated that functional connectivity between the left amygdala and bilateral putamen and right pallidum was negatively correlated with childhood trauma in non-drug using controls. In contrast, functional connectivity between the amygdala and the left middle and inferior frontal cortex, as well as right putamen and caudate, was positively correlated with childhood trauma in regular cocaine users. With other words, in cocaine users, the functional connectivity between the amygdala and dorsal striatum strengthened during the processing of cocaine cues, whereas it reduced during the processing of cocaine cues in non-drug using controls. No such effects were evident for functional connectivity with the right amygdala as seed region (Table 4; Figure 3).

**Table 4:** Differences in amygdala connectivity during cue reactivity (cocaine versus neutral) and the relation to childhood trauma (CTQ), STAI-state, and its interaction.

	Cluster size # voxels	Cluster*p* value	Voxel*z* value	Peak voxel MNI coordinates			Voxel region
**Left amygdala**							
**Group × CTQ**							
Controls—positive correlation^b^	364	0.001	4.08	−24	16	0	L Putamen
			3.89	16	8	0	R Pallidum
			3.64	20	8	−6	R Putamen
Cocaine users—negative correlation^b^	177	0.039	4.43	−22	46	10	L Middle frontal gyrus
			3.53	−32	42	10	L Inferior frontal gyrus
	204	0.023	3.82	26	4	−10	R Putamen
			3.76	14	14	−2	R Caudate
Group × STAI-state	No significant clusters						
**Main effect STAI-state**							
Negative correlation	5,230	<0.001	5.01	16	0	50	R Superior frontal gyrus
			4.85	−48	−4	30	L Precentral gyrus
			4.6	−10	−14	54	L Supplementary motor area
			4.53	56	−12	20	R Postcentral gyrus
			4.36	−60	8	12	L Inferior frontal gyrus
			4.27	−54	−28	48	L Inferior parietal gyrus
			4.24	−50	−16	24	L Postcentral gyrus
	236	0.012	3.94	−50	−48	−6	L Inferior temporal gyrus
			3.65	−54	−46	−4	L Middle temporal gyrus
			3.48	−38	−48	−12	L Fusiform gyrus
	1,884	<0.001	4.26	14	46	18	R Anterior cingulate gyrus
			4.24	10	38	44	R Medial frontal gyrus
			3.93	−16	54	22	L Superior frontal gyrus
			3.93	−4	46	18	L Medial frontal gyrus
			3.81	−10	2	2	L Pallidum
	330	0.002	4.09	−32	28	24	L Inferior frontal gyrus
			3.54	−36	36	22	L Middle frontal gyrus
Positive correlation	No significant clusters						
Group × CTQ × STAI-state	No significant clusters						

**Right amygdala**							
No significant main or interaction effects							

*All results were p < 0.05, cluster level family-wise error corrected with an initial height threshold of p = 0.001 uncorrected*.

*^b^Only those regions that show a significant group by stimulus type interaction effect are reported*.

#### The Relation between Functional Connectivity and State Anxiety

**Figure 4:**
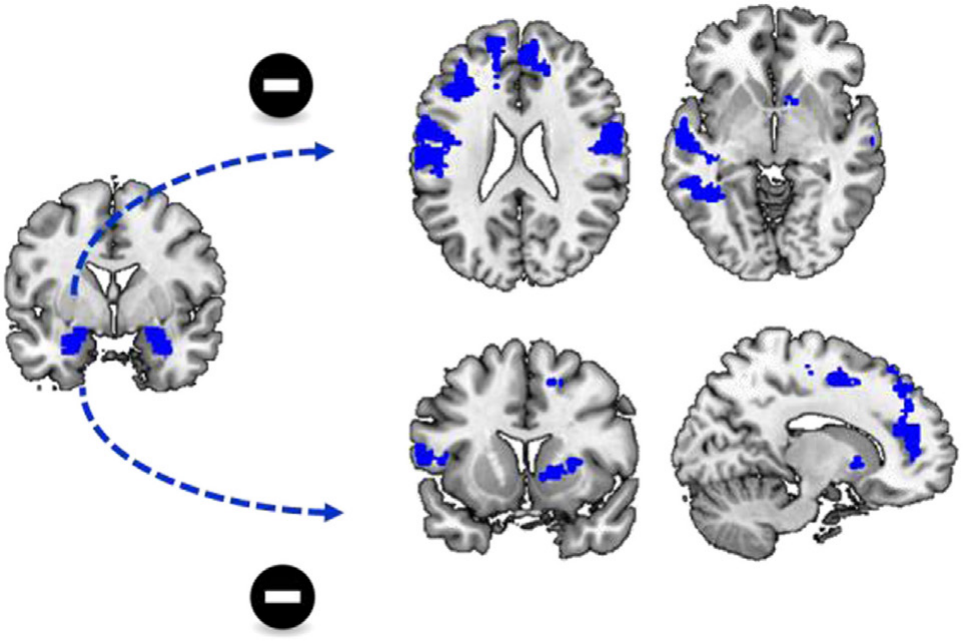
State anxiety and functional connectivity. There was a significant negative correlation between state anxiety and functional connectivity between the left amygdala and a variety of brain regions including the dorsal and ventral medial prefrontal cortex.

For both the left and right amygdala as seed region, there was no significant group by stimulus type by state anxiety interaction effect. however, there was, a negative correlation between state anxiety and functional connectivity of the left amygdala to a variety of brain regions during cocaine cue reactivity Figure 4, Table 4. These regions included the bilateral dorsal medial frontal cortex, the left inferior frontal cortex, and the left inferior and middle temporal cortex. No such effects were evident for functional connectivity with the right amygdala as a seed region (Table 4; Figure 3).

#### The Relation between Functional Connectivity and a Childhood Trauma by State Anxiety Interaction

There was no significant group by stimulus type by CTQ by STAI-state interaction effect.

## Discussion

In this study, we investigated differences in functional connectivity between the amygdala and other brain regions during a cocaine cue reactivity task in male cocaine users and non-drug using controls. In addition, we assessed how these differences were related to childhood trauma and state anxiety. On the neural level, we demonstrated that cocaine cues, compared to neutral cues, elicited stronger activation of the amygdala, nucleus accumbens, and dmPFC and vmPFC in cocaine users only, replicating the findings of several previous studies (37–39). In addition, we demonstrated that cocaine cues, compared to neutral cues, reduced functional coupling between the amygdala (bilaterally) and the dmPFC, inferior frontal cortex, and the insula in addition to some temporal and parietal regions in both cocaine users as non-drug using controls. The amygdala has strong reciprocal connections with the vmPFC and dmPFC, *via* which it can modulate top–down processing (44, 65). Moreover, there was a negative correlation between state anxiety and functional connectivity between the left amygdala and the dmPFC and vmPFC, and dorsal and ventral striatum. The dmPFC, which includes the supplementary motor area, has as a critical role in performance monitoring and cognitive control (66) and has been associated with impaired inhibitory control in addicted individuals (67). The functional coupling between the amygdala and frontal cortex also plays an important role during emotion regulation (48, 68). Hence, impaired amygdala-mPFC functional connectivity may reflect impaired emotional regulation.

The amygdala, in addition to receiving input from frontal cortical regions, projects to widespread striatal domains (44). In this way, the amygdala can modulate striatal output during reward learning and performance (45, 46). It has been suggested that reduced amygdala-striatal connectivity during emotional processing is associated with reduced risk aversion in healthy individuals (50), which may be related to an overreliance on habit behavior (69). Hence, impaired amygdala-striatal functional connectivity is suggested to reflect impaired reward processing.

In contrast to our hypothesis, cocaine cue-related changes in functional connectivity did not differ between cocaine users and non-drug using controls. Therefore, the finding that cocaine cues reduce the function connectivity between the left amygdala and dmPFC and striatum in both cocaine users and controls, and that this is further deteriorated in individuals with high state anxiety, may reflect impaired cognitive control and reduced risk aversion in response to stimuli with a negative emotional valence in general instead of being specific to cocaine cues or individuals with a cocaine use disorder. To assess whether the relation between state anxiety and amygdala-frontostriatal connectivity during cue reactivity reflects general emotional processes or cocaine-specific processes, future studies should include cues with a negative emotional valence in addition to cocaine cues and neutral cues.

On a behavioral level, however, we found that state anxiety was significantly correlated with craving related to negative reinforcement, an effect that has been demonstrated previously in smokers, drinkers, and drug users (19–23). These finding suggest that state anxiety is very relevant in the development, continuation, and treatment of SUDs, as state anxiety may reduce the ability to regulate emotional responses to cocaine-related cues increasing the risk of relapse.

Another important finding of the current study is that a history of childhood trauma is associated with enhanced functional connectivity between the left amygdala and dorsal and ventral striatum in cocaine users, whereas it is associated with reduced functional connectivity between the left amygdala and the dorsal and ventral striatum in non-drug using controls. While reduced amygdala-striatal connectivity has been suggested to underlie reduced risk aversion in healthy participants (50), increased amygdala-striatal connectivity has been previously demonstrated in pathological gamblers (70) and patients with bipolar disorder (71) during reward processing, whereas the control groups in these studies consistently showed reduced amygdala-striatal connectivity during the processing of reward (70, 71). Since the amygdala regulates reward-related signaling in the striatum (72) and inhibiting amygdala-striatal connectivity impairs reward seeking in rodents (73), enhanced amygdala-striatal connectivity during reward processing may underlie impulsive decision making (70). Therefore, a negative correlation between childhood trauma and amygdala-striatum connectivity during cue reactivity in controls and a positive correlation between amygdala-striatum connectivity during cue reactivity in cocaine users suggest that childhood trauma enhances the reward value of cocaine cues in regular cocaine users, whereas it decreases the reward value of cocaine cues in non-drug using controls.

Interestingly, enhanced amygdala-striatal connectivity has also been reported during the processing of negative emotional cues in individuals with a history of childhood trauma (74) and in borderline personality disorder (75). In addition, stress has been shown to increase amygdala-striatum connectivity and stimulus-response learning during memory processing in healthy individuals (76), whereas enhanced amygdala-striatal connectivity predicts poorer cognitive control to emotional cues (49). Hence, an alternative explanation could be that cocaine cues elicit a stress response in cocaine users only, especially in those with a history of childhood trauma, which could underlie enhanced stimulus response or habit behavior as well as poorer cognitive control.

While we cannot draw any conclusions on the causal relation between childhood trauma, amygdala-striatal connectivity, and the development of a cocaine use disorder, various animal studies have demonstrated that early life stress induces serotonergic and dopaminergic changes within the amygdala and striatum (25, 26, 30, 31, 77). Interestingly, the findings of this study suggest that childhood trauma may differentially affect the amygdala-striatal network in individuals at risk versus individuals not at risk for the development of a cocaine use disorder. This hypothesis, however, needs to be addressed using a longitudinal study design.

Interestingly, state anxiety and childhood trauma were specifically related to functional connectivity with the left amygdala as seed region, whereas no effects were found on functional connectivity with the right amygdala as seed region. While these lateralization effects were not expected based on the previous research, emotion regulation is suggested to be primarily associated with left-hemispheric processing of the amygdala and the striatum (78–81). Therefore, the current finding that childhood trauma and state anxiety are specifically related to left amygdala connectivity, which further suggest that these effect mainly reflect altered processes of emotion regulation.

The findings of this study could have clinical implications as the functional coupling between the amygdala and the frontostriatal circuitry may provide us with a novel treatment target. For instance, there is an increasing interest in the use of noradrenergic receptor antagonists in the treatment of alcohol (82, 83) and cocaine (84, 85) use disorder. Interestingly, these receptor antagonists are also suggested to reduce amygdala hyperresponsiveness to negative emotional stimuli (86). Alternatively, repetitive transcranial magnetic stimulation of the prefrontal cortex could be used to alter the functional coupling between the amygdala and frontostriatal circuitry (87). As we demonstrate reduced amygdala-frontostriatal coupling mainly within individuals that report high levels of state anxiety, interventions that target the amygdala and frontostriatal circuitry (including noradrenergic receptor antagonists or rTMS) may be especially effective within these subgroups. On the other hand, interventions that act on amygdala-striatal connectivity may especially be effective in individuals with a cocaine use disorder, with a history of childhood trauma.

The current study has several strengths: first, while the neural correlates of cue reactivity have been extensively studied in alcohol and nicotine use disorder, only a minority of all fMRI cue reactivity studies focused on cocaine use disorder (88). This study, in a relatively large population of cocaine users, therefore adds important novel information to the already existing literature. Second, studying the functional connectivity between the amygdala and frontostriatal circuitry, instead of studying responses within these regions *per se*, is likely to provide a more sensitive measure of neural network function (50, 89).

There are, however, also some limitations. As the majority of the cocaine users included in the current study were polysubstance users, it is unclear whether the findings of the study are specific to cocaine users or whether they reflect alterations specific to alcohol or cannabis users instead. However, as polysubstance use is common among cocaine users in treatment (90), we expect that our sample reflects typical cocaine users. Future studies should be performed to address whether the relation between childhood trauma, state anxiety, and amygdala functional connectivity is differentially effected in cocaine users with and without a history of polysubstance use.

Moreover, there was a strong and positive association between self-reported levels of state anxiety and depressive symptoms. Therefore, we cannot exclude the possibility that amygdala-dmPFC connectivity during cue reactivity is not specifically related to state anxiety, but more generally to negative mood including both depressive and anxiety symptoms. Finally, because cocaine use is much more prevalent among males compared to females, only male participants were included in the study. Gender differences on cue reactivity are scarce, but the few available studies suggest that drug cues induce stronger striatal activity or dopamine release in male compared to female substance users (91–95). Therefore, it remains to be investigated if and how the current findings generalize to female cocaine users, especially when taking into account gender differences in amygdala lateralization (96, 97). Hence, these results need to be replicated in females and other types of substance users as well.

Taken together, our data reveal that childhood trauma is related to enhanced amygdala-striatal connectivity during the processing of cocaine versus neutral cues in individuals with a cocaine use disorder. Enhanced amygdala-striatal connectivity in cocaine users may underlie habit behavior and poorer cognitive control. In addition, we demonstrate that state anxiety is related to reduced amygdala-mPFC connectivity during the processing of cocaine versus neutral cues in both cocaine users as controls. Because reduced amygdala-mPFC connectivity may underlie impaired cognitive control during the processing of stimuli with a negative emotional valence, this may further deteriorate cognitive control in cocaine users. Altogether, these findings provide novel and insight in the neural mechanisms by which childhood trauma and state anxiety may contribute to the development and persistence of cocaine addiction. Eventually this may provide us with novel pharmaceutical or behavioral treatment strategies that target these stress components of cue-induced craving in cocaine users.

## Ethics Statement

The authors assert that all procedures contributing to this work comply with the ethical standards of the relevant national and institutional committees on human experimentation and with the Helsinki Declaration of 1975, as revised in 2008.

## Author Contributions

Data were obtained by AK. AK and GW analyzed the data. The first draft was prepared by AK. JH, WB, LR, and GW actively participated in writing and revising the manuscript for publication.

## Conflict of Interest Statement

The authors declare that the research was conducted in the absence of any commercial or financial relationships that could be construed as a potential conflict of interest. The reviewer KM and handling editor declared their shared affiliation.
